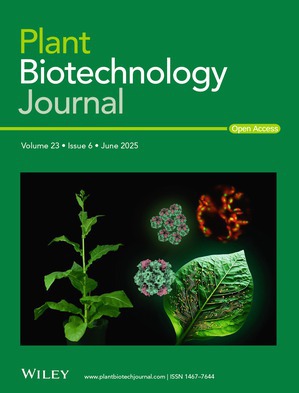# Issue Information

**DOI:** 10.1111/pbi.14392

**Published:** 2025-05-29

## Abstract

Front cover image:

Fusing green fluorescent protein (GFP) to Rubisco results in formation of functional Rubisco condensates within the chloroplasts of engineered tobacco plants, creating dynamic, liquid‐like microcompartments that maintain CO_2_‐fixing activity and plant grow. The study paves the way for employing synthetic biology to enhance photosynthetic efficiency and crop productivity to address climate challenges.

Cover illustration refers to the article published in this issue (Chen et al., pp. 2140–2149).